# Sex Differences in Foot Arch Structure Affect Postural Control and Energy Flow During Dynamic Tasks

**DOI:** 10.3390/life15101550

**Published:** 2025-10-03

**Authors:** Xuan Liu, Shu Zhou, Yan Pan, Lei Li, Ye Liu

**Affiliations:** 1 School of Physical Education, Hubei Normal University, Huangshi 435002, China; liuxuan_12354@163.com (X.L.); zhoushu_tg@163.com (S.Z.); panyan_tg@126.com (Y.P.); 2 School of Sport Science, Beijing Sport University, Beijing 100084, China; lilei120800@163.com

**Keywords:** foot arch, sit-to-stand movement, postural control, energy flow

## Abstract

Background: This study investigated sex differences in foot arch structure and function, and their impact on postural control and energy flow during dynamic tasks. Findings aim to inform sex-specific training, movement assessment, and injury prevention strategies. Methods: A total of 108 participants (53 males and 55 females) underwent foot arch morphological assessments and performed a sit-to-stand (STS). Motion data were collected using an infrared motion capture system, three-dimensional force plates, and wireless surface electromyography. A rigid body model was constructed in Visual3D, and joint forces, segmental angular and linear velocities, center of pressure (COP), and center of mass (COM) were calculated using MATLAB. Segmental net energy was integrated to determine energy flow across different phases of the STS. Results: Arch stiffness was significantly higher in males. In terms of postural control, males exhibited significantly lower mediolateral COP frequency and anteroposterior COM peak velocity during the pre-seat-off phase, and lower COM displacement, peak velocity, and sample entropy during the post-seat-off phase compared to females. Conversely, males showed higher anteroposterior COM velocity before seat-off, and greater anteroposterior and vertical momentum after seat-off (*p* < 0.05). Regarding energy flow, males exhibited higher thigh muscle power, segmental net power during both phases, and greater shank joint power before seat-off. In contrast, females showed higher thigh joint power before seat-off and greater shank joint power after seat-off (*p* < 0.05). Conclusions: Significant sex differences in foot arch function influence postural control and energy transfer during STS. Compared to males, females rely on more frequent postural adjustments to compensate for lower arch stiffness, which may increase mechanical loading on the knee and ankle and elevate injury risk.

## 1. Introduction

The foot arch, as a key anatomical structure for weight support and load distribution, exhibits sex-specific differences in height and stiffness that have been closely linked to injury risk in athletic populations [1,2]. Although women generally present with smaller foot dimensions and a lower degree of pes planus, they nonetheless demonstrate higher rates of musculoskeletal injury than men [3], suggesting that differences in arch height alone are insufficient to explain the sex-specific disparities in injury risk, and that the foot arch is more likely to exert its influence through sex-specific structure–function regulatory mechanisms.

Arch function extends beyond mechanical support of the foot; it may also influence central nervous system modulation of movement strategies and thus affect postural-control stability [4,5]. A recent systematic review and meta-analysis found no significant differences in dynamic balance performance among high-arch, normal, or flat-foot types [6], yet it remains unclear whether sex-related variations in arch structure translate into altered postural-control stability. Energy flow—an index quantifying the efficiency of mechanical-energy transfer between body segments—has been widely employed to assess both performance and injury risk [7,8]. Previous work has shown that augmenting ankle-segment power output during gait can markedly influence proximal lower-limb power and overall energy-flow patterns [9]. However, whether sex-driven arch structural differences, via their impact on postural-control strategies, modulate energy-flow dynamics has yet to be determined.

The sit-to-stand (STS) task—a frequently performed functional movement in daily life—combines elements of postural control with dynamic transition and therefore provides a comprehensive reflection of an individual’s center-of-mass regulation and energy-flow characteristics during weight transfer [10,11]. Accordingly, this study employs the STS paradigm to systematically examine sex differences in arch morphology and function (height and stiffness) and to evaluate how these differences influence postural-control strategies and energy-flow patterns. Elucidating these mechanisms may inform sex-specific training interventions, movement-control assessments, and lower-limb injury-prevention strategies.

## 2. Materials and Methods

A total of 108 healthy adults (53 men and 55 women) participated in the study, with recruitment taking place from 28 April 2024 to 15 June 2024. All participants signed written informed consent and successfully completed the sit-to-stand task. The study protocol was approved by the Ethics Committee of Sports Science Experiments at Beijing Sport University (Approval No. 2024120H).

### 2.1. Participants

This study recruited 108 healthy adults (53 men and 55 women) who met the following inclusion criteria: (1) no absence of sensory or neurological impairments; (2) no prior lower-limb joint surgery; (3) no participation in strenuous exercise within 72 h before testing and no subjective muscle fatigue; (4) no history of systematic or professional sports training. Ethical approval was granted by the Institutional Review Board (Approval No. 2024120H). Participant demographics are summarized in Table 1.

### 2.2. Data Collection

**Figure 1:**
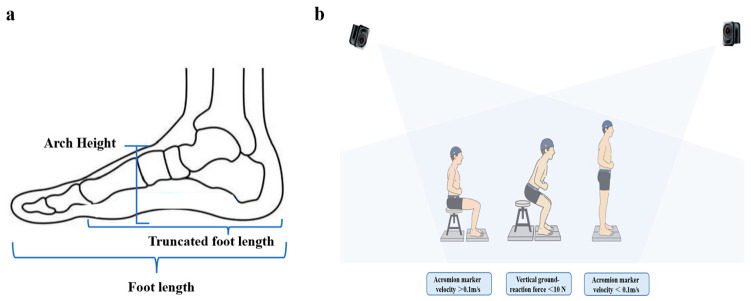
Schematic Illustration of Foot Morphology Measurement and STS Testing. (**a**) Illustration of foot morphological measurement locations; (**b**) Schematic of the STS procedure.

Arch morphology was quantified using a calibrated foot-length measuring device and scale (precision ±0.05 mm). Arch height was defined as the perpendicular distance from the highest point of the navicular tuberosity to the plantar support plane. Foot length was measured from the most posterior aspect of the calcaneus to the distal tip of the longest toe. “Heel-to-first-metatarsal” length was defined as the distance from the posterior calcaneal point to the medial prominence of the first metatarsal head (Figure 1a). This measurement protocol has been widely used in previous studies and has been proven to be highly reliable, ensuring the reproducibility of arch morphology assessment [1,3].

STS trials were performed at the Sport Rehabilitation Medicine Center of Beijing Sport University. Data were collected using an eight-camera infrared motion-capture system (Qualisys, Gothenburg, Sweden; 200 Hz) synchronized with four tri-axial force plates (Kistler, Winterthur, Switzerland; 1000 Hz). Participants wore close-fitting clothing and completed the STS tasks barefoot. Reflective markers were placed by trained staff on standardized anatomical landmarks, including the head, upper limbs, trunk (C7, jugular notch, T10, xiphoid process), pelvis (anterior and posterior superior iliac spines, sacrum), and lower limbs (thighs, medial/lateral femoral condyles, tibial tuberosities, medial/lateral malleoli, heels, and the second and fifth metatarsal heads).

#### 2.2.1. Testing Procedure

Procedure: A backless chair was adjusted so that the seat height equaled 100% of each participant’s shank length, in accordance with our previous work [10]. After maintaining a stable sitting posture for 5 s, the participant stood up at a self-selected speed upon hearing a verbal cue. Each participant performed five valid trials.

#### 2.2.2. Phases of the STS Task

The STS movement was divided into two phases: Pre–seat-off phase: From movement initiation—defined as the instant when the acromion marker velocity exceeded 0.1 m/s—until seat-off, identified by the vertical ground-reaction force under the chair dropping below 10 N. Post-seat-off phase: From seat-off until full trunk extension—defined as the instant when the acromion marker velocity fell below 0.01 m/s.

### 2.3. Data Analysis

The formulas for Arch Height Index (AHI) and Arch Stiffness are defined as follows [12,13]:

AHI = (Dorsum Height/Truncated Foot Length) × 100(1)

Arch Stiffness = (0.4 × Body Weight)/(AHI (seated) − AHI (standing))(2)

Kinematic data were collected using Qualisys Track Manager (QTM, version 2023.3), which was employed to identify the spatial coordinates of reflective markers. The raw trajectories were low-pass filtered using a fourth-order Butterworth filter with a cutoff frequency of 6 Hz. The resulting. C3D files were imported into Visual3D (version 4.0, C-Motion Inc., Germantown, MD, USA) to construct a full-body rigid segment model and to compute the center of pressure (COP) and center of mass (COM).

To minimize the influence of morphological differences among participants, the time-series data were normalized as follows: COP and COM trajectories in the anterior–posterior (AP) direction were normalized to foot length; mediolateral (ML) trajectories were normalized to the distance between the left and right anterior superior iliac spines (ASIS); and vertical (V) COM trajectories were normalized to body height [14].

Postural control metrics were defined as follows [12,13,15]. Maximum displacement: The largest deviation of the COP or COM from a reference point, reflecting the extent of postural sway. Maximum velocity: The peak instantaneous velocity in a given direction, indicating the system’s rapid reactive capability in postural adjustment. Frequency: The mean oscillation frequency of the COP signal, representing the rhythmic characteristics of postural control. Sample entropy: A measure of the complexity and regularity of time-series data. Higher values of sample entropy indicate more irregular and complex sway patterns, suggesting reduced postural stability. Sample entropy was calculated following the method proposed by Richman et al. [16], using a custom-written MATLAB script (version 2022a), with an embedding dimension of m = 2 and a tolerance threshold of r = 0.2. Momentum: The mean linear momentum of the COM, representing the inertial behavior of whole-body movement. Larger momentum values imply greater difficulty in maintaining postural control.

Energy flow variables were processed following the procedures described in our previous work [10]. Joint power (*JP*) was defined as the rate of work performed by joint forces on adjacent segments. A positive value indicates passive energy absorption by the segment, whereas a negative value reflects energy output or generation. Here, *Fj* represents the joint force and *vj* the linear velocity at the joint center. Power describes the rate of energy transfer between segments per unit time. The total energy flow of a segment includes both active and passive contributions at its proximal and distal ends [16]. Passive power flow results from joint reaction forces and translational velocities, while active power flow is generated by muscle work. Positive segment power denotes energy input into the segment, and negative segment power indicates energy leaving the segment [9,17].

Joint Power (*JP*): Quantifies passive energy transfer into (positive) or out of (negative) adjacent segments.

(3) JP=Fj⋅νj

Muscle Power (*MP*): Calculated as the product of joint torque (*Mj*) and segment angular velocity (*ωs*).

(4) MP=Mj⋅ωs

Segment Power (*SP*): represents net energy exchange within a segment, computed as the sum of distal/proximal joint power (*JPd* and *JPp)* and muscle power (*MPd* and *MPp)*.

(5) SP=JPd+JPp+MPd+MPp

### 2.4. Statistical Analysis

The normality of all variables, including arch height index, arch stiffness, postural control measures, and energy flow parameters, was tested using the Shapiro–Wilk test. Variables meeting the normality assumption were compared between groups with independent-samples *t*-tests, while non-normally distributed data were analyzed using the Mann–Whitney U test. Statistical significance was defined as *p* < 0.05. All analyses were conducted in MATLAB R2024a (The MathWorks, Natick, MA, USA).

## 3. Results

### 3.1. Sex Differences and Correlation Analysis of AHI and Arch Stiffness

**Figure 2:**
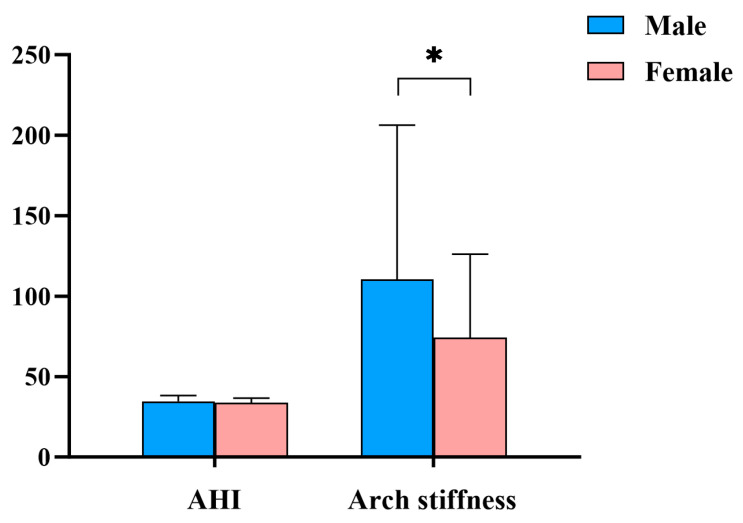
Sex Differences Between AHI and Arch Stiffness. Notes: Values are presented as mean ± SD. Error bars indicate SD. * indicates a statistically significant difference between males and females (*p* < 0.05).

As shown in Figure 2, arch stiffness was significantly higher in males compared to females (*p* = 0.017).

### 3.2. Sex Differences in Postural Control During the STS

**Figure 3:**
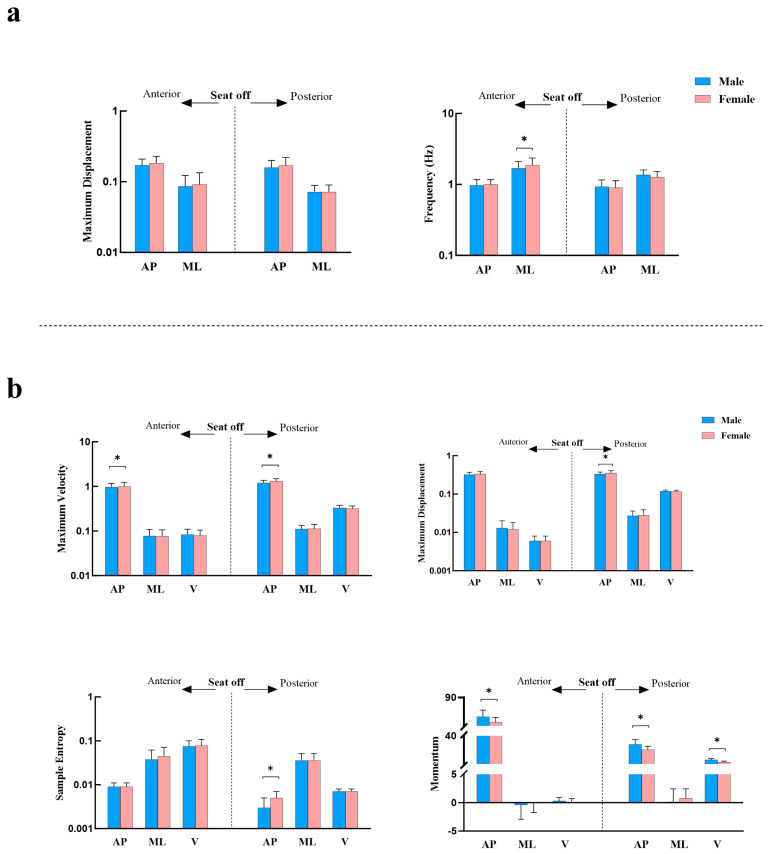
Sex Differences in COP and COM During the STS. (**a**) Sex differences in COP-related metrics during the pre- and post-seat-off phases of the STS; (**b**) Sex differences in COM-related metrics during the pre- and post-seat-off phases of the STS. Notes: Values are presented as mean ± SD. Error bars indicate SD. * indicates a statistically significant difference between males and females (*p* < 0.05).

As shown in Figure 3, during the pre-seat-off phase of the STS task, females exhibited a higher mediolateral COP frequency (*p* = 0.045) and a higher maximum anteroposterior COM velocity (*p* = 0.025) compared to males, while their anteroposterior COM momentum was significantly lower (*p* < 0.001). In the post-seat-off phase, females demonstrated greater maximum anteroposterior COM displacement (*p* = 0.047), higher maximum velocity (*p* = 0.004), and significantly higher sample entropy (*p* < 0.001) than males. However, their anteroposterior and vertical COM momentum values remained significantly lower than those of males (*p* < 0.001).

### 3.3. Sex Differences in Energy Flow During the STS

**Figure 4:**
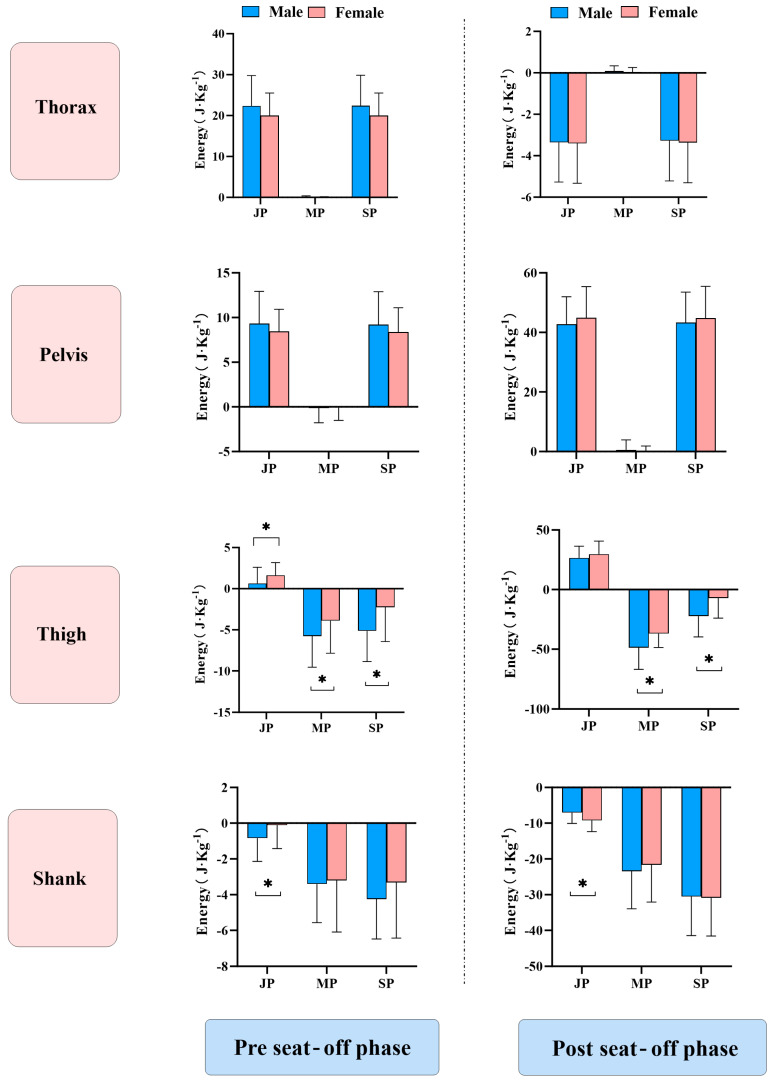
Sex Differences in Energy Flow During the STS. Notes: Values are presented as mean ± SD. Error bars indicate SD. * indicates a statistically significant difference between males and females (*p* < 0.05).

As illustrated in Figure 4, during the pre-seat-off phase of the STS task, females exhibited higher hip joint power compared to males (*p* = 0.005), whereas muscle power (*p* = 0.013) and net segment power (*p* < 0.001) were lower in females. In the post-seat-off phase, females showed significantly lower hip muscle power (*p* < 0.001) and net segment power (*p* < 0.001) than males, while calf joint power was significantly higher in females (*p* < 0.001).

## 4. Discussion

This study investigated sex differences in the structure and function of the foot arch, as well as how these differences influence postural control and energy flow strategies during dynamic tasks, thereby elucidating the sex-specific functional mechanisms of foot arch characteristics. Our results revealed that although males and females exhibit similar arch structure, their functional performance differs, with males demonstrating greater arch stiffness. Furthermore, during dynamic tasks, males and females employed distinct postural stability and energy flow control strategies.

Previous studies have reported that males exhibit greater arch stiffness [17], which may be attributed to their wider metatarsal arch span and the higher Young’s modulus of the Achilles tendon and plantar fascia, resulting in a stronger mechanical response. In contrast, females may present lower arch stiffness due to relatively weaker muscle strength and greater ligamentous laxity [18]. Consistent with these findings, the present study also demonstrated that, despite comparable arch height indices between sexes, males exhibited significantly higher arch stiffness.

While earlier studies in adolescents have suggested minimal sex differences in postural stability [19], our findings demonstrate significant sex-related differences in postural control during dynamic movements in adults. Females exhibited higher sample entropy and postural adjustment frequency during the STS, potentially reflecting the need for more frequent dynamic adjustments to compensate for lower arch stiffness and maintain balance. Males showed lower mediolateral COP frequency and maximum anteroposterior COM velocity in the pre-seat-off phase, as well as reduced maximum COM displacement, velocity, and sample entropy in the post-seat-off phase compared to females. These results suggest that males adopt a lower-frequency, more regular postural adjustment pattern during STS, indicative of greater postural control “stability.” This enhanced stability may be attributed to increased foot load response capacity conferred by higher arch stiffness, combined with greater momentum gain [20], facilitating more efficient mechanical transmission and center-of-mass transfer [21]. However, this stability might come at the expense of dynamic flexibility, potentially increasing the risk of overload-related injuries such as anterior knee pain [22]. Notably, these findings do not contradict the higher epidemiological prevalence of anterior knee pain in females. On the contrary, our results suggest that the relatively greater knee joint loading and reduced ankle–knee energy transfer efficiency observed in females may lead to elevated patellofemoral joint stress, thereby partially explaining their greater susceptibility to anterior knee pain despite lower absolute power output.

Regarding energy flow, males exhibited significantly higher hip muscle power and net segment power during STS, likely due to stronger quadriceps muscles acting as the primary driver in this task. In contrast, females showed distinct joint power distribution patterns: calf joint power was significantly lower than males during the pre-seat-off phase but higher post-seat-off; additionally, the power transferred from the calf to the thigh before seat-off was significantly greater in females. Previous research has indicated that imbalances in active and passive energy flow within the kinetic chain can elevate injury risk [7,23]. Our findings suggest that during STS, females experience increased distal lower-limb joint loading as the center of mass rises, with heightened knee joint loading early in the movement phase due to its role as the link between the thigh and calf. This may be attributed to lower arch stiffness in females, leading to reduced ankle–knee mechanical response efficiency at the onset of dynamic tasks, thereby increasing inter-joint mechanical loading and elevating the risk of patellofemoral pain syndrome, patellar tendinopathy, and other overuse-related knee injuries.

From a practical standpoint, our findings provide evidence to inform sex-specific training and injury-prevention strategies. For females, interventions should focus on strengthening intrinsic foot muscles, improving ankle–knee mechanical response efficiency, and incorporating closed-chain lower-limb exercises to reduce patellofemoral joint stress. For males, training programs may emphasize improving dynamic adaptability and ankle mobility to distribute loads more evenly and mitigate the risk of stiffness-related overuse injuries.

Limitations: This study did not include muscle strength assessments or surface electromyography analyses, limiting insights into sex-specific neuromuscular control strategies. In addition, the sample consisted exclusively of healthy young adults, which may limit the generalizability of the findings to other age groups or individuals with musculoskeletal conditions; future studies should extend to other age groups and clinical populations to enhance the clinical relevance and translational potential of these findings.

## 5. Conclusions

There are significant sex differences in foot arch function, which in turn affect postural control strategies and energy flow patterns during the STS. Compared to males, females rely on more frequent postural adjustments to compensate for lower arch stiffness. While this helps maintain stability, it also increases mechanical load on the knee and ankle joints and may elevate the risk of injury.

## Figures and Tables

**Table 1:** Characteristics of the participants (Mean ± SD).

	Male	Female
Age (y)	22.3 ± 2.35	22.71 ± 2.51
Height (cm)	176.12 ± 5.39	164.01 ± 5.41
Weight (kg)	70.37 ± 9.95	55.67 ± 5.87
Body mass index (kg/m^2^)	22.61 ± 2.46	20.72 ± 1.72
Foot length (cm)	25.56 ± 1.14	23.24 ± 0.89
Truncated foot length (cm)	20.75 ± 1.43	22.2 ± 21.43
Standing arch height (cm)	7.14 ± 0.59	6.47 ± 0.53
Sitting arch height (cm)	7.61 ± 0.65	6.92 ± 0.56

## Data Availability

The raw data are available from the corresponding author upon reasonable request.
